# LncRNA and transcriptomic analysis of fetal membrane reveal potential targets involved in oligohydramnios

**DOI:** 10.1186/s12920-020-00792-z

**Published:** 2020-09-18

**Authors:** Yu-hua Ou, Yu-kun Liu, Li-qiong Zhu, Man-qi Chen, Xiao-chun Yi, Hui Chen, Jian-ping Zhang

**Affiliations:** 1 Department of Obstetrics and Gynecology, Guangdong Women and Children Hospital, Guangzhou, 511400 Guangdong China; 2 Department of Obstetrics and Gynecology, Sun Yat-sen Memorial Hospital, Sun Yat-sen University, No.107, Yanjiangxi Road, Guangzhou, 510120 Guangdong China

**Keywords:** Biological process, Regulatory network, Fetal membrane, Oligohydramnios

## Abstract

**Background:**

The multiple causes of oligohydramnios make it challenging to study. Long noncoding RNAs (lncRNAs) are sets of RNAs that have been proven to function in multiple biological processes. The purpose of this study is to study expression level and possible role of lncRNAs in oligohydramnios.

**Methods:**

In this study, total RNA was isolated from fetal membranes resected from oligohydramnios pregnant women (OP) and normal amount of amniotic fluid pregnant women (Normal). LncRNA microarray was used to analyze the differentially expressed lncRNAs and mRNAs. Kyoto Encyclopedia of Genes and Genomes (KEGG) was used to analyze the main enrichment pathways of differentially expressed mRNAs. Real-time quantitative PCR (qPCR) was used to validate the lncRNA expression level.

**Results:**

LncRNA microarray analysis revealed that a total of 801 lncRNAs and 367 mRNAs were differentially expressed in OP; in these results, 638 lncRNAs and 189 mRNAs were upregulated, and 163 lncRNAs and 178 mRNAs were downregulated. Of the lncRNAs, 566 were intergenic lncRNAs, 351 were intronic antisense lncRNAs, and 300 were natural antisense lncRNAs. The differentially expressed lncRNAs were primarily located in chromosomes 2, 1, and 11. KEGG enrichment pathways revealed that the differentially expressed mRNAs were enriched in focal adhesion as well as in the signaling pathways of Ras, tumor necrosis factor (TNF), estrogen, and chemokine. The qPCR results confirmed that LINC00515 and RP11-388P9.2 were upregulated in OP. Furthermore, the constructed lncRNA–miRNA–mRNA regulatory network revealed tenascin R (TNR), cystic fibrosis transmembrane conductance regulator (CFTR), ATP-binding cassette sub-family A member 12 (ABCA12), and collagen 9A2 (COL9A2) as the candidate targets of LINC00515 and RP11-388P9.2.

**Conclusions:**

In summary, we revealed the profiles of lncRNA and mRNA in OP. These results might offer potential targets for biological prevention for pregnant women with oligohydramnios detected before delivery and provided a reliable basis for clinical biological treatment in OP.

## Background

Amniotic fluid is critical for a healthy pregnancy because it allows for fetal movements and it protects the fetus from trauma by acting as a physical cushion. It also plays an important role in fetal lung and limb development [1]. The volume of amniotic fluid varies at different stages of pregnancy [2, 3], and the average amniotic fluid volume is 400 mL at term [4].

Oligohydramnios is generally defined as a reduced amount of amniotic fluid. Amniotic fluid volume in the third trimester of pregnancy with less than 300 mL, amniotic fluid index (AFI) < 5 cm, and single deepest pocket (SDP) ≤ 2 cm [5–8] are the commonly used parameters for diagnosis. Oligohydramnios is a common complication during pregnancy; it can increase delivery rates and labor induction rates in pregnant women and can significantly increase the mortality rate of perinatal children [9, 10]. In addition, oligohydramnios is associated with intrauterine fetal growth restriction and prolonged labor [11].

Currently, the mechanisms underlying oligohydramnios remain unclear. Long noncoding RNA (lncRNA) is a class of transcripts that contains more than 200 nucleotides, but it cannot encode proteins. LncRNAs have been proven to be expressed in a wide range of diseases, and they are involved in regulating cancer development and metastasis [12], heart diseases [13, 14], and autoimmune diseases [15]. There is also evidence suggesting that lncRNA is involved in pregnancy-associated events. For example, placental lncRNA expression is alert in response to phthalate exposure during pregnancy [16]. Furthermore, lncRNA uc003fir suppresses the proliferation and migration of trophoblast cells, which might contribute to preeclampsia development [17]. However, little is known about the association between lncRNAs and oligohydramnios.

Therefore, in the present study, we performed lncRNA and mRNA microarray analyses to explore the lncRNA and mRNA expression profile in response to oligohydramnios in pregnant women. Both lncRNAs and mRNAs were sequenced for lncRNA–miRNA–mRNA integrated analysis. In this study, we provided the first evidence that lncRNAs and mRNAs were differentially expressed in the fetal membrane in oligohydramnios pregnant women (OP); based on the lncRNA–miRNA–mRNA network, we predicted the potential role of lncRNAs and mRNAs in OP.

## Methods

### Patient recruitment

We conducted a retrospective cohort study of pregnant women with oligohydramnios in Sun Yat-sen Memorial Hospital. In 2017, the number of deliveries in the Obstetric Department of Sun Yat-sen Memorial Hospital was 2667. Among them, 45 cases were pregnant women with oligohydramnios. Due to emergency cesarean section of pregnant women, part of fetal membrane tissues was lost due to failure to freeze in liquid nitrogen within 15 min. Finally, the fetal membrane tissues of 20 pregnant women with oligohydramnios and 19 normal amount of amniotic fluid pregnant women (Normal) were collected and used in this study. The age of all pregnant women was between 21 and 37 years. Both groups of pregnant women had no secondary diseases, intrauterine infection, smoking, alcohol, fetal developmental abnormalities, acute chorioamnionitis, premature rupture of membranes, and drugs used, including angiotensin converting enzyme inhibitor, angiotensin II receptor blockers, non-steroidal anti-inflammatory drugs during pregnancy. The blood pressure in the two groups of pregnant women was within the normal range.

### Diagnostic criteria for oligohydramnios

Pregnant women who meet the following standard criteria are diagnosed with oligohydramnios: amniotic fluid volume in the third trimester of pregnancy with less than 300 mL, an SDP of ≤2 cm or an AFI of ≤5 cm [5–8]. Simultaneously, when the AFI is less than 8 cm, it is considered to be less amniotic fluid volume [18]. The mean values of the AFI and SDP for pregnant women with oligohydramnios, which were detected and diagnosed by the same sonographer through ultrasound and the pregnant women with oligohydramnios were re-examined by ultrasound again at the internal of 2–4 days, were 53.81 ± 13.82 and 26.95 ± 7.51 mm, respectively. And the content of amniotic fluid estimated by the obstetrician in pregnant women with oligohydramnios during delivery was about 194.29 ± 50.06 mL, which was less than 300 mL. The fetal membrane tissues of pregnant women with AFI ≤ 5 cm were used for microarray analysis. The fetal membrane tissues of pregnant women with AFI of 53.81 ± 13.82 mm and SDP of 26.95 ± 7.51 mm were used for real-time quantitative PCR (qPCR) verification. Simultaneously, the obstetric outcomes (Table 1) and pregnancy complications (Table 2) of pregnant women were also analyzed.

**Table 1:** Demographic information

Characteristics	Oligohydramnios (***n*** = 20)	Normal control (***n*** = 19)	P-value
Age (years)	32.63 ± 5.23	30.8 ± 4.17	0.233
Weeks of delivery	37.34 ± 2.01	39.84 ± 2.88	0.003*
BMI	26.21 ± 2.48	26.23 ± 2.81	0.977
Number of pregnancy	2.48 ± 1.50	2.68 ± 1.62	0.68
Number of productions	1.38 ± 0.50	1.47 ± 0.88	0.695
Total number of abortions	1.09 ± 1.34	1.21 ± 1.15	0.775
Spontaneous abortion	0.16 ± 0.37	0.7 ± 0.92	0.023*
IVF (%)	2 (9.5)	1 (5.2)	0.538
Apgar 1 min	9.65 ± 0.74	9.89 ± 0.31	0.131
Baby gender			
Male	8	8	–
Female	12	11	–
Delivery method			0.072
Normal delivery (%)	8(4)	13(68.4)	
Cesarean section (%)	12(60)	6(31.6)	
Birth weight (kg)	2.67 ± 0.57	3.31 ± 0.23	3.89E-5*
Placental weight (kg)	0.47 ± 0.04	0.51 ± 0.02	0.003*

*BMI* Body Mass Index, *IVF* In-vitro fertilization. *P < 0.05 (Student’s t-test). Variable is mean ± standard deviation

**Table 2:** Pregnancy complications

Characteristics	Oligohydramnios (n = 20)	Normal control (n = 19)	P-value
FGR (%)	7(35)	0	0.005^#^
RSA (%)	5 (25)	0	0.027^#^
Premature birth (%)	6 (30)	0	0.012^#^
GDM (%)	1 (5)	2 (10.5)	0.48
UCTD (%)	1 (5)	0	0.513
Mild anemia (%)	2 (10)	0	0.256
Hypothyroidism (%)	2 (10)	4 (21.1)	0.305
Thalassemia (%)	2 (10)	0	0.256

*FGR* Fetal growth restriction, *RSA* Recurrent abortion, *GDM* Gestational diabetes mellitus, *UCTD* Undifferentiated connective tissue disease. *P < 0.05 (Student’s t-test). Classification variable is calculated by ratio (%). ^#^Fisher was used to accurately test *P*-value < 0.05

### Tissue collection

When giving birth in an operating room or a maternity room, after the placentas were delivered, the fetal membrane tissues 2 cm from the periphery of the umbilical cord were cut. The cut fetal membrane tissues, which were washed with sterile phosphate buffered saline (PBS) and then dried with sterile gauze, were about 4 cm*4 cm in size. All samples were immediately frozen in liquid nitrogen within 15 min and then transferred to − 80 °C refrigerator for storage for later use.

### RNA isolation

The membranes were washed prior to being homogenized. Approximately 1 cm^3^ of the tissue block was resected for grinding. Samples were ground in a motor-driven homogenizer. Trizol (Invitrogen, CA, USA) was used to extract total RNA from the tissues in accordance with the manufacturer’s protocol. The concentration and qualification of the isolated total RNA was assessed by a Nanodrop 2001 spectrophotometer (Thermo Fisher Scientific, MA, USA).

### LncRNA and mRNA microarray analysis

Total RNA from the fetal membranes, which were obtained from five OP and five Normal, were used for microarray analysis. The Human LncRNA Array V4.0 (8 × 60 k) was performed by KangChen Bio-tech Inc. (Shanghai, China) according to the manufacturer standard protocols. The microarray analyses included 40,173 lncRNAs and 20,730 mRNAs.

### Bioinformatics

Agilent Feature Extraction software (version 11.0.1.1) was used to analyze the acquired array images. Quantile normalization and subsequent data processing were performed with the GeneSpring GX v11.5.1 software package (Agilent Technologies). Differentially expressed lncRNAs and mRNAs between two conditions were identified through fold change filtering. Heatmaps and scatter plots were generated for differentially expressed genes using the R package (version 3.1.0) [19]. Kyoto Encyclopedia of Genes and Genomes (KEGG) pathway analysis was performed using an online tool (http://www.genome.jp/kegg/). KEGG pathways that met the requirement of False Positive Discovery (FDR) ≤ 0.001 were considered significantly enriched.

To explore the potential role of lncRNA, a lncRNA–miRNA–mRNA interaction network was constructed. We used miRNA target gene prediction software (miRanda) to predict miRNA targets on lncRNA. The overlap miRNAs that harbored both lncRNA and mRNA binding targets were used to construct the lncRNA–miRNA–mRNA interaction network. The sub-network that contained predicted targets of lncRNA and was differentially expressed in OP was included. The network was visualized using Cytoscape_V2_8_3 (https://www.innatedb.ca/cytoscape-v2.8.3/plugins/) software.

### qPCR

The relative expression of lncRNA between 20 OP and 19 Normal was measured by qPCR. Total RNA was reverse transcribed to cDNA using PrimeScript RT Master Mix (Takara, Dalian, China). cDNAs were then amplified and quantified on an ABI 7500 real-time PCR system (Applied Biosystems, CA, USA) with a SYBR Real time PCR Master Mix Kit (TOYOBO, Osaka, Japan). The program for cDNA amplification was as follows: the first step, 95 °C for 120 s; the second step, 95 °C for 15 s and 60 °C for 30 s, for 40 cycles; the third step, for melting curve generation, 60 °C to 95 °C. The relative expression of lncRNA was analyzed using the 2^−ΔΔCt^ method. GAPDH was used as an internal control. The primers were shown in Table 3.

**Table 3:** Primers used in this study

Primer name	Sequence (5′ to 3′)	Production size
LINC00515F	TCAAGGCAGCAGTGGCAGAG	142
LINC00515R	AGTCACAGGCGTGGAGGTCA	
RP11-388P92F	ATTTGCCAGCTTCTCCTTTGA	145
RP11-388P92R	TTGGCAGAATGAGACATCAAG	
GAPDHF	GAGTCAACGGATTTGGTCGT	185
GAPDHR	GAGTCAACGGATTTGGTCGT	

### Statistical analysis

Student’s t-test was used to analyze the significant differences by the SPSS 18.0 software package in the study. Three biological replicates were performed in the study. A *P*-value of < 0.05 defined the significant differences between the two groups.

## Results

### Overview of lncRNA and mRNA profiles in OP

There was no significant difference in the age, number of pregnancy, and number of productions between the two groups of pregnant women (Table 1). The weeks of delivery and number of spontaneous abortion in the OP were 37.34 ± 2.01 and 0.7 ± 0.92, respectively, which were significantly lower than those of Normal group (Table 1). The main delivery methods in OP and Normal groups were cesarean section (60%) and normal delivery (68.4%), respectively (Table 1). The birth weight and placental weight in OP group were 2.67 ± 0.57 and 0.47 ± 0.04 kg, respectively, which were significantly lower than those of the Normal group (Table 1). Some pregnant women with oligohydramnios had symptoms of gestational diabetes mellitus (GDM), undifferentiated connective tissue disease (UCTD), mild anemia, hypothyroidism, and thalassemia (Table 2). However, there were only 2 cases of gestational diabetes mellitus and 4 cases of hypothyroidism in the Normal group (Table 2).

**Fig. 1:**
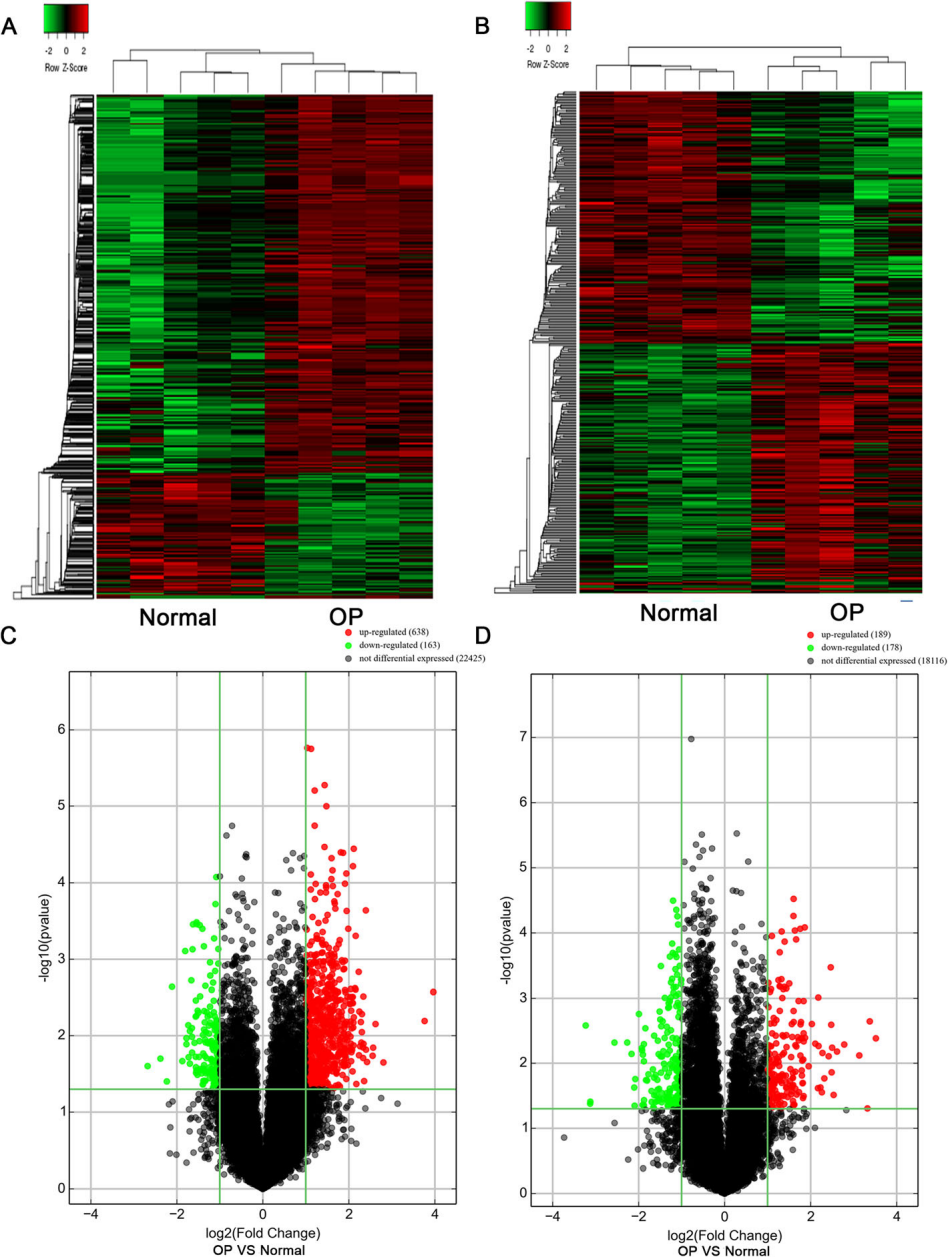
Microarray analysis revealed differential lncRNA profiles in fetal membranes resected from five oligohydramnios pregnant women (OP) and five normal amount of amniotic fluid pregnant women (Normal). The heatmap showed the profile of lncRNA (**a**) and mRNAs (**b**) in OP and Normal. The volcano plot showed the overall change in expression of lncRNAs (**c**) and mRNAs (**d**). The upregulated RNA is labeled in red, whereas the downregulated RNA is labeled in green

To explore the profile of lncRNA and mRNA in the fetal membranes of five OP and five Normal were obtained and subjected to microarray analysis. As shown in Fig. 1, the profiles of lncRNA and mRNA in OP were different from those in Normal. A sequence of lncRNAs and mRNAs were alert in OP, compared with Normal (Fig. 1a&b). Among the differentially expressed lncRNAs, 638 were upregulated and 163 were downregulated (Fig. 1c and Supplemental Table 1). Of the differentially expressed mRNAs, 189 were upregulated and 178 were downregulated, as shown in the volcano plot (Fig. 1d and Supplemental Table 2).

### Characteristics of the differentially expressed lncRNAs and mRNAs in OP

**Fig. 2:**
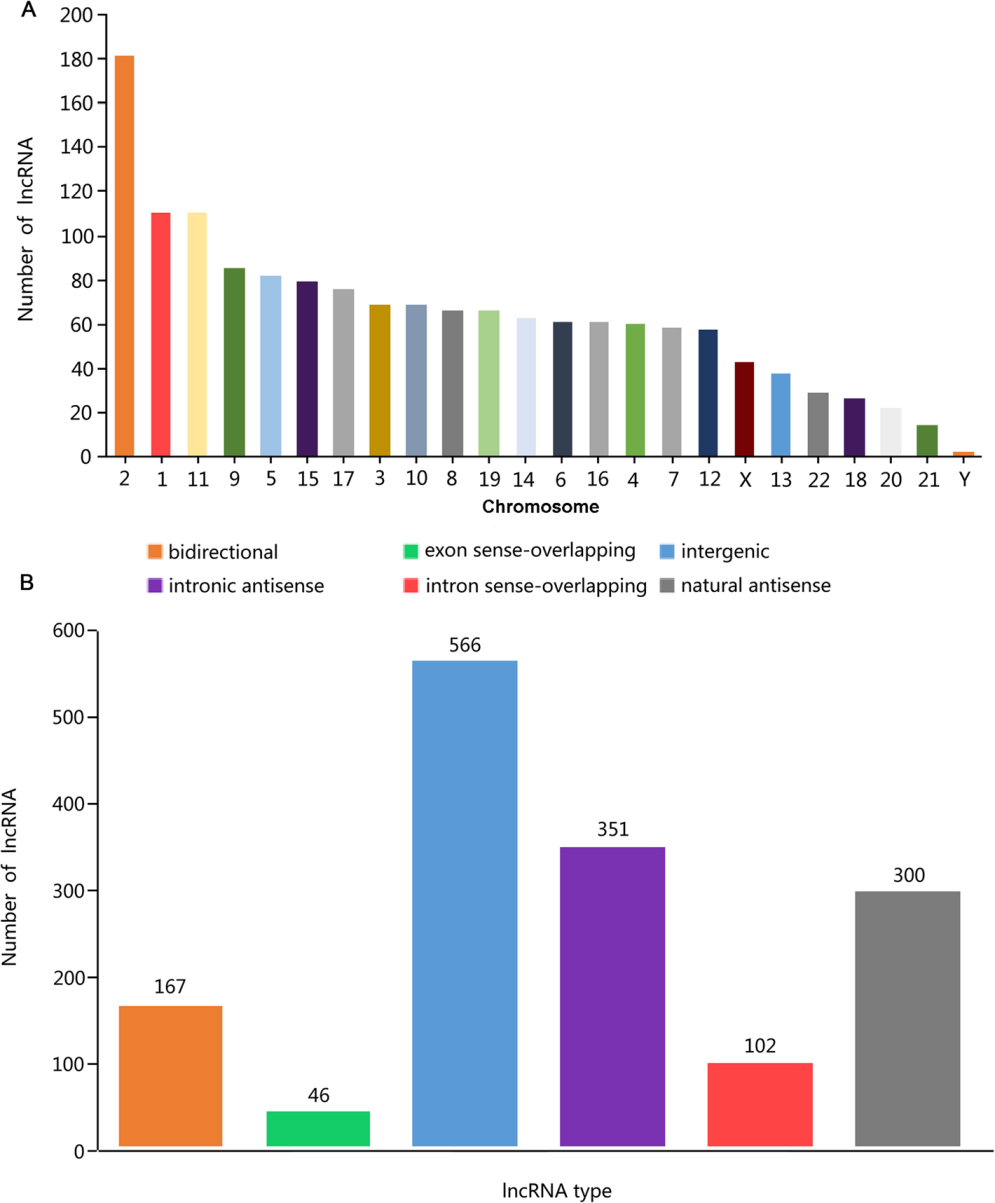
Distribution of lncRNA genomic location (**a**) and type of the differentially expressed lncRNA (**b**) in oligohydramnios pregnant women (OP)

**Fig. 3:**
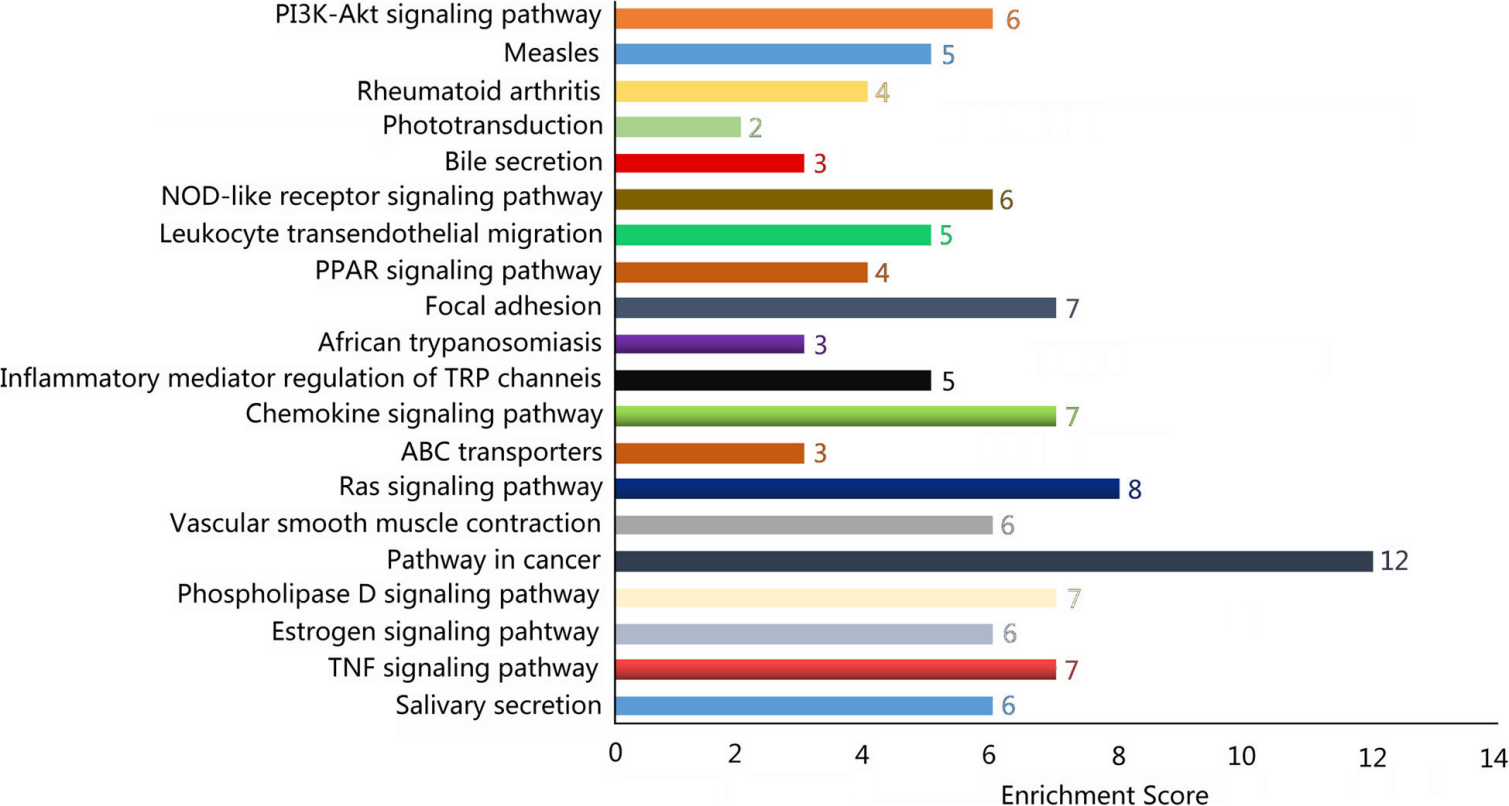
Kyoto Encyclopedia of Genes and Genomes (KEGG) enrichment analysis showing the most enriched pathways for the differentially expressed mRNAs in oligohydramnios pregnant women (OP)

To further observe the expression characteristics of these differentially expressed lncRNAs and mRNAs, there was an analysis of the genomic location distribution as well as the length and type distribution. The statistical results of the differentially expressed lncRNA showed that the differentially expressed lncRNA was mainly distributed on chromosomes 2, 1, and 11, with the least distribution on the Y chromosome; this indicated that the lncRNAs that played a role in oligohydramnios were mainly located on chromosomes 2, 1 and 11 (Fig. 2a). The length distribution showed that most lncRNAs were distributed within 1 kb, whereas the mRNAs were mainly distributed at 2–3 kb in length (Supplemental Figure 1). Analysis for differential lncRNA type revealed the largest number of intergenic lncRNAs, followed by intronic antisense and natural antisense, indicating that the lncRNAs that played a role in oligohydramnios are mainly intergenic lncRNAs (Fig. 2b). KEGG results revealed that differential mRNAs mainly enrich in focal adhesion as well as in the signaling pathways of Ras, tumor necrosis factor (TNF), estrogen, and chemokine (Fig. 3). Other top pathways were shown in Fig. 3.

### The expression of lncRNAs and the potential regulatory network

**Fig. 4:**
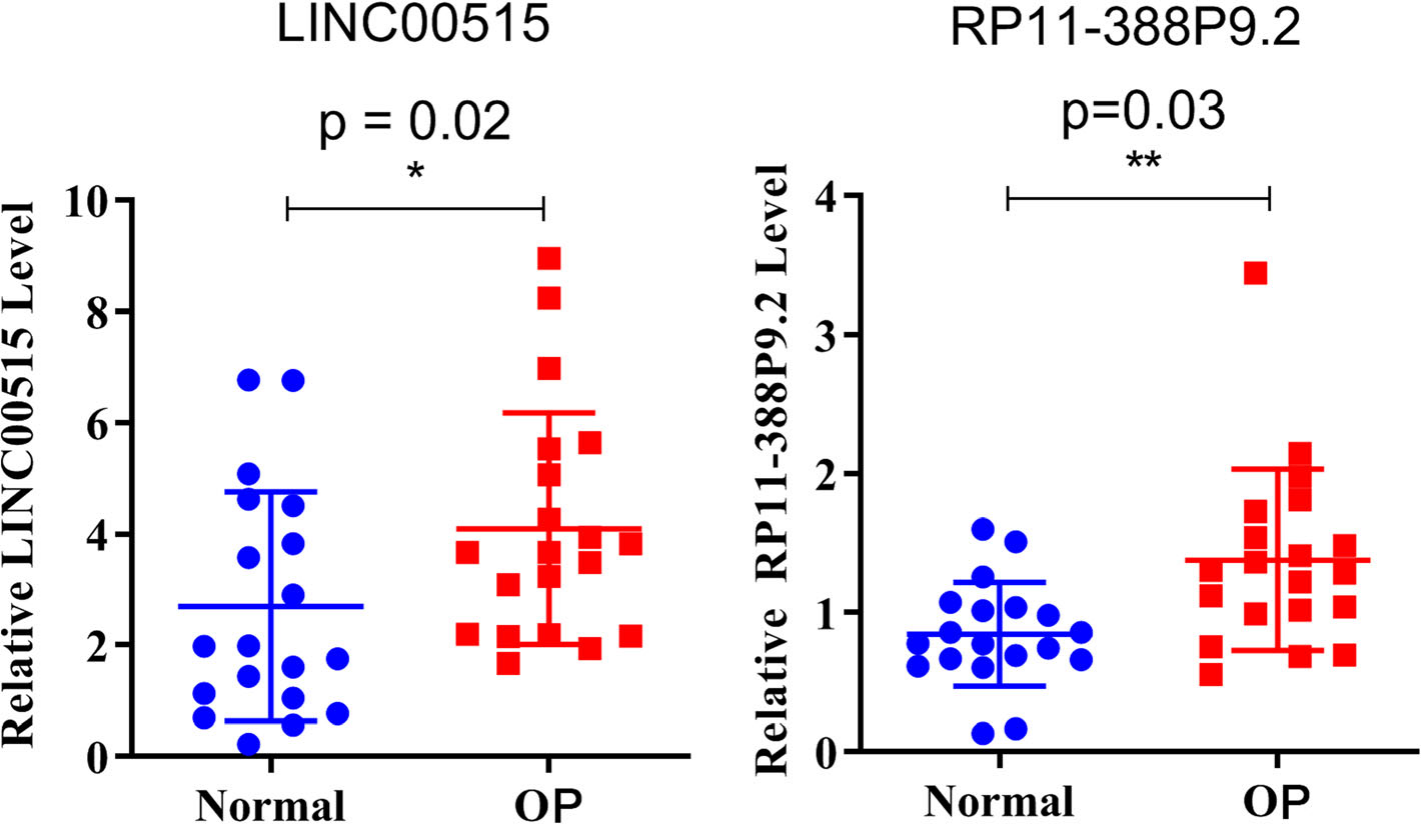
The expression of LINC00515 and RP11-388P9.2 in oligohydramnios pregnant women (OP) and normal amount of amniotic fluid pregnant women (Normal). *P < 0.05 represents the significant difference

**Fig. 5:**
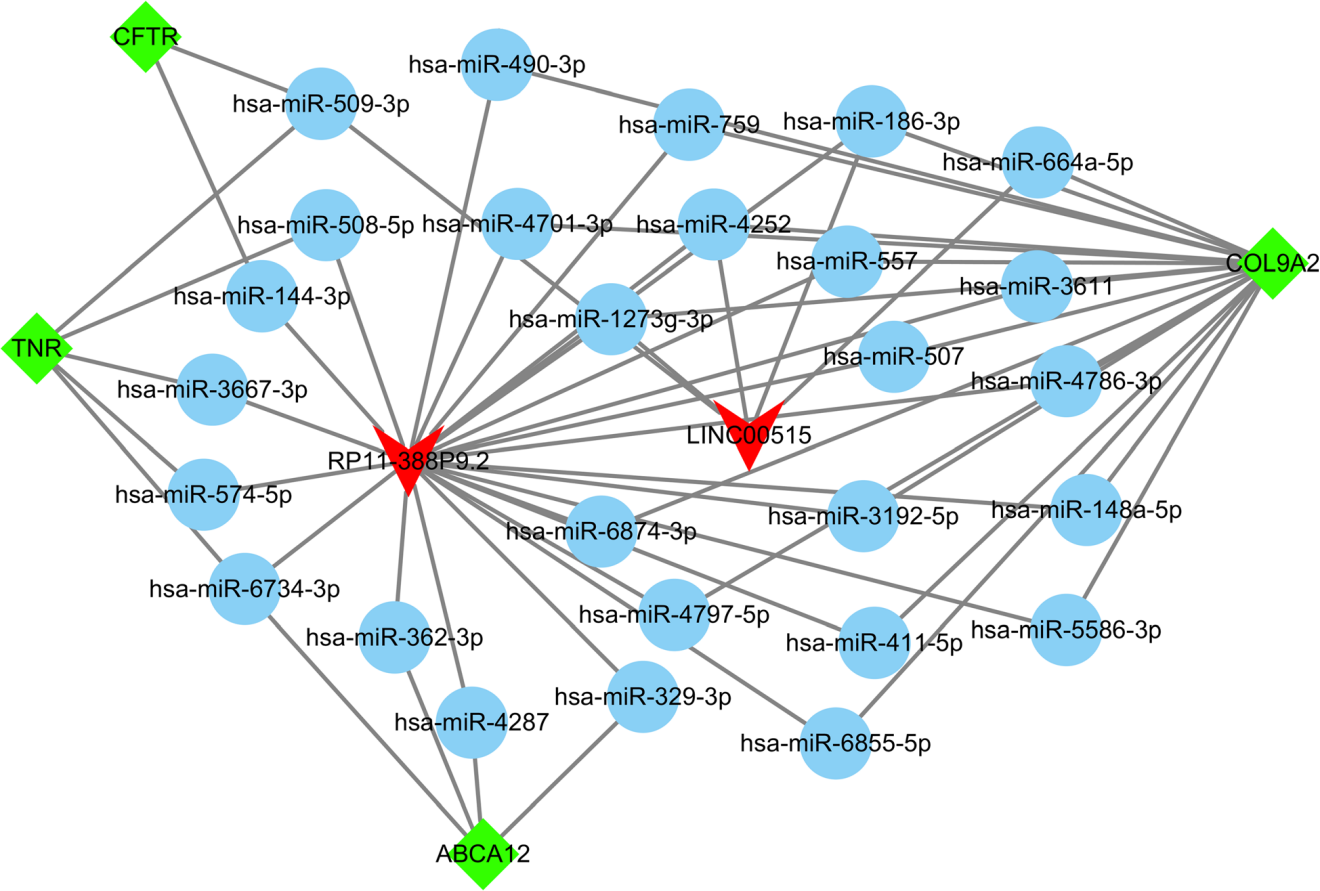
The lncRNA–miRNA–mRNA interaction network. The miRNAs are also potential targets of LINC00515 and RP11-388P9.2

In order to explore the function of differentially expressed lncRNAs in OP, specific analysis was conducted for the differentially expressed lncRNAs and their regulatory network. Table 4 listed the lncRNAs in the top 10 of upregulated and downregulated expression in OP women. As shown in Table 4, the highest differential expression was G017197, and the upregulation fold change was 6.99 times; the highest downregulation was G083088, and the expression was downregulated to 0.15-fold. We verified the differential expression of two specific lncRNAs, LINC00515 and RP11-388P9.2, by using qPCR (Fig. 4). As shown in Fig. 4, both LINC00515 and RP11-388P9.2 showed increased expression in OP. Furthermore, a lncRNA–miRNA–mRNA interaction network based on LINC00515 and RP11-388P9.2 was generated. Potential miRNA targets of LINC00515 and RP11-388P9.2 were predicted and then screened for consistency with mRNAs that were expressed as upregulated in OP. As revealed in Fig. 5, a regulatory network of LINC00515 and RP11-388P9.2 was obtained. The network included 27 miRNAs and 5 mRNAs (Fig. 5). The mRNAs that were finally captured were tenascin R (TNR), cystic fibrosis transmembrane conductance regulator (CFTR), ATP-binding cassette sub-family A member 12 (ABCA12), and collagen 9A2 (COL9A2).

**Table 4:** Information of the top-10 most upregulated and downregulated lncRNAs in oligohydramnios pregnant women (OP)

lncRNA_ID	Fold Change	Regulation	P-value	FDR
G017197	6.992997551	up	0.022330367	0.323992752
G028960	6.156767122	up	0.007011989	0.231007727
G056426	5.925028116	up	0.014297859	0.282223415
GSE61474_XLOC_033346	5.875790672	up	0.018248847	0.30550168
G059353	5.369031987	up	0.022024259	0.322125587
AC091729.7	5.280611483	up	0.015183552	0.286476988
LINC00515	5.265016308	up	0.000229591	0.092301652
G024752	5.123561472	up	0.039183093	0.394165858
RP11-388P9.2	5.0709114	up	0.01273173	0.271540086
DPP10-AS1	5.0609152	up	0.003061877	0.185019347
G083088	0.156292992	down	0.024924406	0.335128165
LINC00501	0.192207162	down	0.019901778	0.31149042
G045291	0.213060492	down	0.039570275	0.395804997
LINC01510	0.231630157	down	0.002294572	0.176676707
RP11-1399P15.1	0.273437307	down	0.021416751	0.321541985
RP11-150O12.1	0.286446378	down	0.000781865	0.143720717
LRRC74B	0.292328015	down	0.010723383	0.257909493
RP11-567G11.1	0.292488539	down	0.012877268	0.272737873
G016402	0.301039562	down	0.007261346	0.232753219
G043293	0.302661694	down	0.019071938	0.309549149

*FDR* False positive discovery

## Discussion

Oligohydramnios is one of the common obstetric complications. The etiology of oligohydramnios mainly includes the fetal factor, placental membrane factor, maternal factor, and drug factor. For example, in the prolonged pregnant women with oligohydramnios, resistance index in the fetal renal artery is higher than it is in the controls, which are without oligohydramnios [20]; additionally, cyclooxygenase-2 inhibitor nimesulide and long-term diclofenac exposure are associated with oligohydramnios [21, 22]. In our study, the possible causes of oligohydramnios in pregnant women may be due to an increased number of abortions and pregnancy complications; still, the underlying molecular mechanism for oligohydramnios remains unknown. Therefore, we conducted microarray analysis and unveiled the expression profile of lncRNA and mRNA in OP. The qPCR results confirmed that LINC00515 and RP11-388P9.2 were upregulated in OP. lncRNA–miRNA–mRNA regulatory network revealed TNR, CFTR, ABCA12, and COL9A2 as the candidate targets of LINC00515 and RP11-388P9.2. These results showed that the expression of lncRNAs in fetal membrane tissues might plays an important role in pregnant women with oligohydramnios.

The fetal membrane is a vital tissue for communication between mother and fetus. There is a hypothesis that the resorption pathway that crosses the amnion to the fetal circulation may keep the balance of normal amniotic fluid volume [20]. Therefore, it is reasonable to speculate that changes in the expression of molecules in the membrane tissue are response to changes in the microenvironment. Many studies have previously provided the molecular information [23–26] of the fetal membrane tissue. The expression of aquaporin 8 and aquaporin 9 in amniotic membrane of oligohydramnios group is significantly lower than that of normal amniotic fluid group, however, it has not been shown which lncRNAs regulate the expression of aquaporin 8 and aquaporin 9 in oligohydramnios group [27]. Simultaneously, the expression analysis of lncRNA and mRNA in the fetal membrane tissue of pregnant women with oligohydramnios is still limited. Here we showed that 638 lncRNAs and 189 mRNAs were upregulated and that 163 lncRNAs and 178 mRNAs were downregulated. Moreover, we found that the differentially expressed mRNAs were mainly enriched in focal adhesion as well as in the signaling pathways of Ras, TNF, estrogen, and chemokine. Research shows that TNF-α and interleukin 6 (IL-6) play an essential role in the inflammatory process in pregnant women with spontaneous preterm births [28]. Under the induction of ferutinin, human amniotic fluid stem cells are differentiated into osteoblasts through estrogen receptor α and the phosphatidylinositol 3-kinase (PI3K)/protein kinase B (Akt) signal pathway [29]. The TNF signaling pathway and the estrogen signaling pathway may be involved in the regulation of OP.

We also confirmed the upregulation of two lncRNAs, LINC00515 and RP11-388P9.2, in OP. Also, a lncRNA–miRNA–mRNA interaction network was constructed to illustrate the possible function of LINC00515 and RP11-388P9.2 involved in oligohydramnios. In the network, the role of almost all miRNAs in oligohydramnios remains unknown. In addition, four mRNAs, CFTR, TNR, ABCA12, and COL9A2 were predicted to be included in the regulatory network of LINC00515 and RP11-388P9.2. Of these mRNAs, CFTR, a small conductance Cl^−^ channel, is regulated by intracellular ATP and cAMP-dependent phosphorylation, predominantly located in the apical membrane of organ epithelial cells. The study of endometrial epithelia shows that CFTR is involved in the secretory effects of ovarian hormone regulation [30], showing that CFTR plays a vital role in female reproduction. It can be inferred that RP11-388P9.2 might affect the CFTR expression by regulating has-miR-114-3p, further affecting the amniotic fluid content of pregnant women. Moreover, TNR can interact with fibronectin 1, which can be involved in cell migration and adhesion biological processes including embryogenesis [31]. Tenascin is also produced in the extracellular matrix of cultured amnion epithelial cells [32]. It can be inferred that RP11-388P9.2 might affect the TNR expression by regulating the has-miR-508-5p, has-miR-3667-3p, has-miR-574-5p, and has-miR-6734-3p, further affecting the amniotic fluid content of pregnant women. Compared with the amniotic fluid cells of a healthy human, the cells of fetuses with neural tube defects do not deposit type I collagen [33]. It can be inferred that LINC00515 might affect the COL9A2 expression by regulating the has-miR-186-3p and has-miR-664a-5p, further affecting the amniotic fluid content of pregnant women.

Based on the above deduction, we could conclude that LINC00515 and RP11-388P9.2 regulated the expression of different miRNAs to control the expression levels of TNR, CFTR, ABCA12, and COL9A2, which affected the decrease of amniotic fluid content in pregnant women. However, their mechanism is still unclear and needs further study.

## Conclusions

In summary, we revealed the profiles of lncRNA and mRNA in OP, validated the upregulation of LINC00515 and RP11-388P9.2, and suggested a lncRNA–miRNA–mRNA network that might be involved in the pathogenesis of oligohydramnios. These results might offer potential targets for biological prevention for pregnant women with oligohydramnios detected before delivery.

## Supplementary information

**Additional file 1: Figure S1.** Distribution of lncRNA length in oligohydramnios pregnant women (OP).

**Additional file 2: Table S1.** Differentially expressed lncRNAs between group-normal amount of amniotic fluid pregnant women (Normal) and the group-oligohydramnios pregnant women (OP).

**Additional file 3: Table S2.** Differentially expressed mRNAs between group-normal amount of amniotic fluid pregnant women (Normal) and the group-oligohydramnios pregnant women (OP).

## Data Availability

The datasets used and analyzed during the current study are available from the corresponding author on reasonable request. The datasets generated during the current study are available in the GEO Database Series GSE142701 repository, [https://www.ncbi.nlm.nih.gov/geo/query/acc.cgi?acc=GSE142701].

## Abbreviations

LncRNAs: Long noncoding RNAs
OP: Oligohydramnios pregnant women
Normal: Normal amount of amniotic fluid pregnant women
KEGG: Kyoto Encyclopedia of Genes and Genomes
qPCR: Real-time quantitative PCR
TNF: Tumor necrosis factor
TNR: Tenascin R
CFTR: Cystic fibrosis transmembrane conductance regulator
ABCA12: ATP-binding cassette sub-family A member 12
COL9A2: Collagen 9A2
AFI: Amniotic fluid index
SDP: Single deepest pocket
qPCR: Real-time quantitative PCR
PBS: Phosphate buffered saline
RPKM: Reads per kilobase of transcript per million reads mapped
FDR: False Positive Discovery
GDM: Gestational diabetes mellitus
UCTD: Undifferentiated connective tissue disease
IL-6: Interleukin 6
PI3K: Phosphatidylinositol 3-kinase
Akt: Protein kinase B
