# Supplementary figures and images for: LncRNA and transcriptomic analysis of fetal membrane reveal potential targets involved in oligohydramnios
Source: BMC Med Genomics. 2020 Sep 18;13:137. doi: 10.1186/s12920-020-00792-z (PMC7501699; doi:10.1186/s12920-020-00792-z)

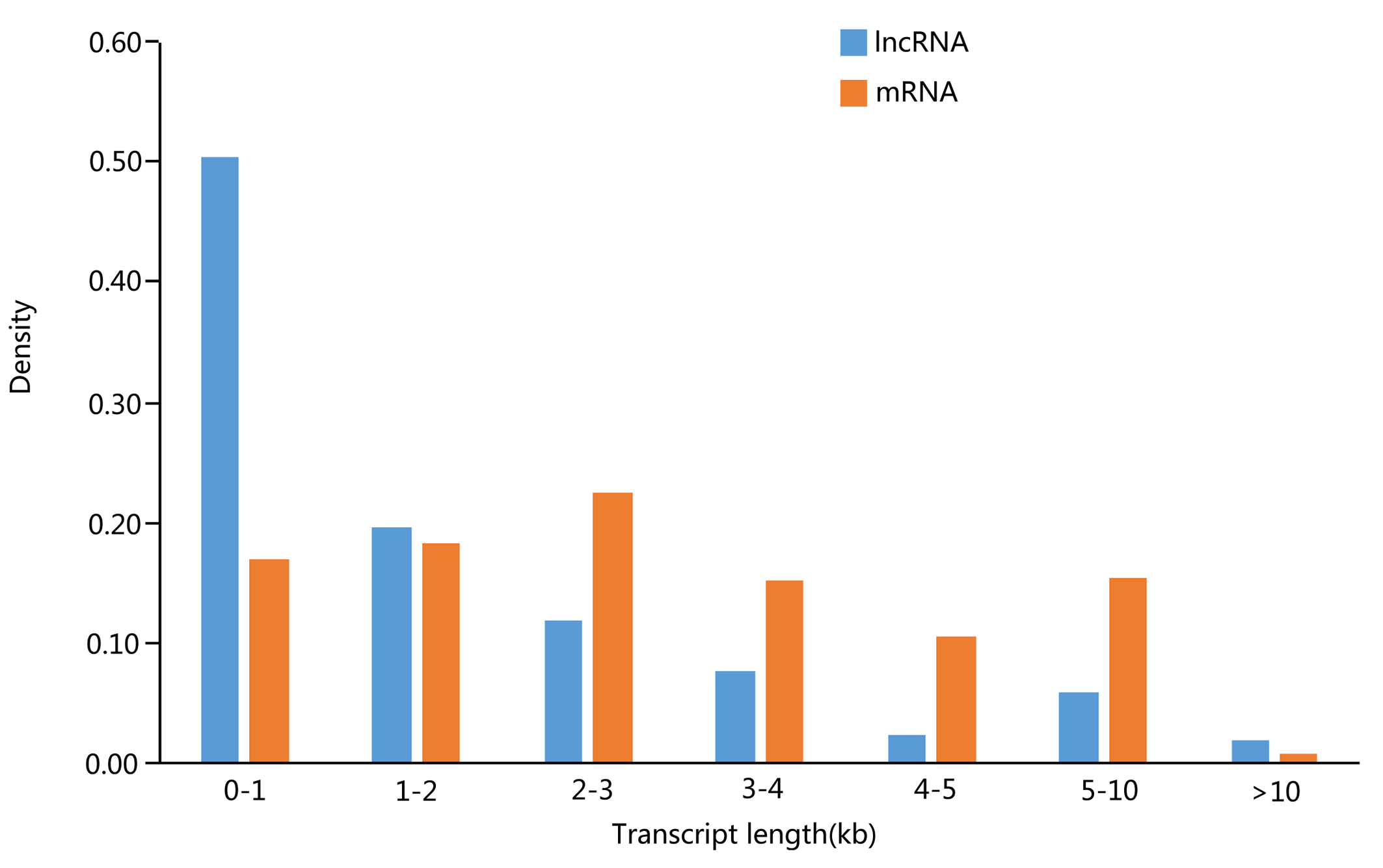

Supplement: Supplementary file 1 — Additional file 1: Figure S1. Distribution of lncRNA length in oligohydramnios pregnant women (OP). [file 12920_2020_792_MOESM1_ESM.tif]
